# Glioma: interaction of acquired and germline genetics

**DOI:** 10.18632/aging.203428

**Published:** 2021-08-12

**Authors:** Jeanette E. Eckel-Passow, Daniel H. Lachance, Robert B. Jenkins

**Affiliations:** 1 Department of Quantitative Health Sciences, Mayo Clinic, Rochester, MN 55905, USA; 2 Department of Laboratory Medicine and Pathology, Mayo Clinic, Rochester, MN 55905, USA; 3 Department of Neurology, Mayo Clinic, Rochester, MN 55905, USA

**Keywords:** glioma, GWAS, polygenic, IDH, CCDC26

Adult diffuse gliomas can be molecularly classified into more homogenous subtypes with similar clinical and molecular features using two acquired molecular alterations: *IDH* mutation and 1p/19q codeletion. Starting in 2016 and continuing in the 2021 edition, the WHO criteria integrated traditional histopathologic assessment with these two acquired alterations for pathological diagnosis of glioma [1]. The three primary molecular resulting subtypes are tumors with (i) both *IDH* mutation and 1p/19q codeletion, which are now referred to as “oligodendroglioma, *IDH*-mutant, and 1p/19q codeleted”, (ii) tumors with *IDH* mutation and with 1p/19q intact, which are now referred to as “astrocytoma, *IDH*-mutant”, and (iii) *IDH* wildtype tumors, which are now referred to as glioblastoma, *IDH*-wildtype. Current research has been aimed at further understanding these molecular subtypes, including the germline variants that are associated with development of these molecular subtypes.

Initial genome-wide association studies (GWAS) that treated glioma as a single entity identified nine variants in eight genes that were associated with development of adult diffuse glioma [2]. Subsequently, two GWAS performed by histological subtype identified 18 additional novel germline variants: six that were associated specifically with high grade glioma (grade IV, glioblastoma) and 12 that were associated with low grade glioma (grade II-III) [2,3]. Notably, these variants only reached genome-wide significance when the GWAS was performed within these two histological subtypes. More recently, our team performed a GWAS using the 2016 WHO criteria, stratifying patients by *IDH* mutation and 1p/19q codeletion and identified two additional novel regions: SNPs in *D2HGDH* were associated with tumors that had an *IDH* mutation and a SNP near *FAM20C* was associated with tumors that had both *IDH* mutation and 1p/19q codeletion [4].

The observed germline associations likely reflect the progression of glioma development. For example, while most variants are associated with particular histologic or molecular subtypes, the *TP53* germline variant is associated with the development of all gliomas. Thus, *TP53* may interact with some germline (or acquired) variants to facilitate the development of *IDH*-mutant glioma, and other germline (or acquired) variants to facilitate the development of *IDH* wild-type glioma (primary glioblastoma). Overall, it is very interesting that many of the germline variants associated with glioma risk are within or near genes that are commonly altered in brain tumors (e.g., *CDKN2A/B*, *TERT*, *EGFR*, *IDH1*, etc.).

One particularly interesting germline variant is rs55705857, which is associated with an approximate 6-fold increased risk of developing an *IDH* mutated glioma and is similar in effect size as *BRCA1* with breast cancer risk. Rs55705857 is located within an intron of *CCDC26*, on chromosome band 8q24.21, a region that contains very few protein-coding genes. While the 8q24.21 region is associated with development of many cancers, most variants in this region that are associated with other cancers are approximately 1.5 Mb centromeric to rs55705857 [5]. The rs55705857 variant is associated with age at glioma diagnosis, with patients carrying the risk G allele having a significantly younger age at diagnosis compared with patients with the non-risk allele [6,7].

The germline associations can also be used to calculate a polygenic risk score, from which to estimate relative and absolute risk of overall glioma and glioma subtypes. Using the known glioma germline variants, we developed a polygenic risk model and observed that patients with a risk score in the highest 5% for *IDH*-mutant 1p/19q codeleted subtype or for *IDH*-mutant 1p/19q intact subtype had more than a 14-fold increased risk of developing a glioma with an *IDH* mutation in comparison to patients with median risk scores [8]. These risk scores will not be used for population screening because of the low lifetime risk of developing glioma. However, we are currently evaluating the clinical utility of these risk scores in pre-defined high-risk groups. It is important to acknowledge that the genetic studies discussed above were all performed in European populations and thus the estimated risks may not be applicable to other populations. More research is necessary to performed GWAS in more diverse populations.

Now that the major germline variants associated with glioma risk have been identified, it is critical that functional genomic studies be performed to discern the mechanism of why these variants are associated with glioma development. There will likely be important mechanistic relationships between the germline variants and the various acquired molecular alterations that are observed in the tumors and it is likely that the interplay between germline and acquired alterations will have important clinical and biologic significance.
